# Simultaneous Abdominal Wall Reconstruction and Panniculectomy in High-Risk Complex Patients: A Retrospective Cohort Study

**DOI:** 10.3389/jaws.2025.15456

**Published:** 2025-12-17

**Authors:** Antoine Vancon, Fahad Alhammadi, Alix Donadieu, Pierre Gueroult, Reza Kianmanesh, Quentin Delecroix, Yohann Renard

**Affiliations:** 1 Department of General, Digestive and Endocrine Surgery, Christian Cabrol University Hospital, University of Reims Champagne-Ardenne, Reims, France; 2 Department of Anatomy, Faculty of Medicine, University of Reims Champagne-Ardenne, Reims, France; 3 Department of Plastic Surgery, Christian Cabrol University Hospital, University of Reims Champagne-Ardenne, Reims, France; 4 LICIIS Laboratory, LRC DIGIT URCA-CEA, University of Reims Champagne-Ardenne, Reims, France

**Keywords:** ventral hernia, panniculectomy, abdominal wall reconstruction, loss of domain, component separation

## Abstract

**Introduction:**

Ventral hernia repair (VHR) in high-risk, complex obese patients often presents a technical challenge for surgeons. Performing a panniculectomy (PAN) at the same time as the VHR may be an acceptable option for enhancing hernia exposure, improving cosmetic outcomes, and improving subsequent quality of life (QoL). However, this approach remains controversial due to the theoretical increase in postoperative complications. The objective of this study was to evaluate the relevance of combining PAN with VHR in complex obese patients in a specialised centre in France.

**Materials and Methods:**

A retrospective single-arm study was conducted, including all patients who underwent VHR with PAN from January 2020 to December 2024 at a high-complexity referral hernia centre in Reims, France. Preoperative, intraoperative, and postoperative data were collected and analysed. Complications were categorised according to the Surgical Site Occurrences (SSOs), Surgical Site Infections (SSIs) and Clavien-Dindo classifications. Emergency hernia repairs were excluded.

**Results:**

A total of 45 patients were included. The maximum median BMI was 40.26 kg/m^2^. All patients had at least one comorbidity, more than half had a loss of domain or a stoma, and more than 70% were classified as 2 or 3 according to the VHWG classification. The component separation technique was necessary for 42% of patients. The SSO and SSI rates were 58% and 7%, respectively, with an unplanned reoperation rate of 15%. After a median follow-up of 31 weeks, 11% of patients developed a hernia recurrence.

**Conclusion:**

Combining PAN with VHR in complex obese patients increases the risk of minor wound complications but does not significantly affect the rate of major complications or recurrence. PAN may introduce additional morbidity for complex VHR in obese patients and should be considered in selected cases after a thorough risk-benefit evaluation.

## Introduction

Approximately 4 million laparotomies are performed annually in the United States [1], 500,000 in France [2]. Incisional hernias are therefore a common outcome, affecting 12.8%–30% of patients [3], and resulting in a significant physical impact, poor quality of life [4, 5] and chronic pain [6]. Ventral hernia repair (VHR) itself is a common surgical procedure, with approximately 600,000 cases performed annually in the United States and 40,000 in France [2, 7]. The socio-economic burden is substantial, as VHR is estimated to cost nearly €70 million/year in France and $9 billion/year in the United States [8, 9]. These figures may be an underestimation, given the high rates of recurrence and postoperative complications associated with VHR [10].

Obesity is an important independent risk factor for both the development of incisional hernias and their recurrence after VHR [6, 11]. Moreover, obesity increases the risk of postoperative complications, including surgical-site occurrences (SSOs) and infections (SSIs), along with the need for reintervention [10]. As nearly 45% of the global population is now overweight or obese [12, 13], VHR has become increasingly challenging, underscoring the need for innovative approaches to achieve durable repairs with acceptable outcomes [14].

Obesity is also frequently associated with complex ventral hernias, characterised by larger hernia sacs, multiorgan involvement, denervation, loss of domain (LOD) and multiple comorbidities [15]. These factors make surgical management more difficult and often justify referral to a specialised high-volume hernia centre [16].

In overweight patients with significant abdominal adiposity, the combination of ventral hernia repair (VHR) and panniculectomy (VHR-PAN) as a single-stage procedure is increasingly being considered as a viable option [6]. This combined approach may offer several advantages. Surgically, it can improve exposure and facilitate the complex reconstruction techniques that are often required for obese patients [17]. Additionally, excess skin with poor perfusion and potentially contaminated scar tissue can be removed, thereby reducing the risk of skin necrosis, SSOs and SSIs [13, 17, 18]. Quilting sutures may further secure the reconstruction [19]. By reducing flap tension on the abdominal wall, the panniculectomy may potentially minimise wound-related complications that frequently lead to reoperation [19]. Beyond surgical considerations, long-term patient satisfaction may derive from both cosmetic and functional benefits, including improved hygiene, mobility, body image, and overall quality of life1 [1, 6, 14, 18, 20].

Nevertheless, the safety and efficacy of VHR-PAN remain debated due to concerns about increased wound morbidity and prolonged operative times when both procedures are performed simultaneously [1, 14, 17, 19].

Evidence on this topic remains conflicting. While a 2020 systematic review and meta-analysis suggested a potential reduction in SSIs [21], more recent studies have suggested comparable [11, 22] or worse [13, 14] outcomes when VHR-PAN is performed compared to VHR alone. For example, the most recent systematic review, from 2024, reported increased wound morbidity, a higher need for reoperation, and longer hospital stays in the combined group [17]. Similarly, an analysis of more than 55,000 patients from the American Hernia Database revealed a 57% SSO rate in VHR-PAN *versus* 40% for VHR alone and a twofold increase in unplanned reoperation [13, 18]. The long-term recurrence rate after VHR-PAN is rarely reported in the literature but appears to be similar to that observed after VHR alone [14, 17, 21, 22]. Notably, these studies also report higher SSO rates in VHR-PAN groups, despite SSOs being a recognised risk factor for recurrence [23]. This discrepancy may be partly explained by the fact that the majority of VHR-PAN procedures are performed in specialised, high-volume centres with dedicated expertise in complex abdominal wall reconstruction, where optimised perioperative management may help mitigate the risk of recurrence despite worse SSO rates.

Overall, performing panniculectomy concurrently with VHR remains a widely discussed topic [18], as no clear evidence establishes the superiority of one approach over the other, and the advisability of performing both procedures simultaneously remains questioned [1]. Given that postoperative complications in high-risk, complex obese patients are already common [24–26], reaching up to 80% in some studies [27], the additional morbidity attributable to PAN may be relatively low.

The present study aimed to report on the impact of simultaneous VHR-PAN in a high-volume, single-surgeon, specialised centre for complex incisional hernia surgery in France. This study was based on a retrospective cohort of complex obese patients who were operated on over a period of more than 5 years.

## Materials and Methods

The medical records of all consecutive patients who underwent PAN in combination with (VHR), including both incisional and primary ventral hernias, at the Reims Abdominal Wall Center (RAWC) between 1 January 2020 and 31 December 2024 were retrospectively reviewed. RAWC is a specialised hernia centre affiliated with the University Hospital of Reims, France. Patients younger than 18 years ol or with a prior history of PAN were excluded. Emergency hernia repairs were also excluded.

Demographic data collected included sex, age, physical activity, Body Mass Index (BMI) and relevant comorbidities, namely smoking status, hypertension (HTA), diabetes mellitus and chronic obstructive pulmonary disease (COPD). Hernia history was also recorded.

Hernia width and length were documented. In cases of LOD, calculated according to the Tanaka index [28], patients were managed preoperatively with a standardised protocol, including Botulinum toxin A injection (BTA) and progressive pneumoperitoneum (PPP), as required by our institutional guidelines [29]. The risk of SSOs was evaluated according to the Modified Ventral Hernia Working Group (VHWG) classification [30].

Regarding the surgical technique, the type of PAN was documented (horizontal, *Fleur-de-Lis*, or vertical) and performed by the plastic surgeon. The weight of the resected panniculus was recorded. Details about the mesh were recorded, including mesh type and placement in the plane. The use of surgical drains, the duration of surgery and whether a component separation technique (CST) was performed were also documented. A standard postoperative wound dressing was placed.

Postoperative assessment within 30 days included surveillance for surgical site infections (SSIs), defined as any infection involving the superficial, deep or organ/space layers. SSOs were defined as the occurrence of any of the following: SSIs, seroma, wound dehiscence, cellulitis, a non-healing incisional wound, skin necrosis, an abscess, or a haematoma. Postoperative complications were additionally graded using the Clavien–Dindo classification system.

The final follow-up was defined as the last documented abdominal clinical examination of the patient.

Continuous variables were described as medians (interquartile range 25–75 [IQR]) and qualitative variables were described as frequencies (percentages). This descriptive, single-arm study aimed to report outcomes in a high-risk and complex cohort without comparing them to VHR alone.

## Results

A total of 46 patients underwent VHR-PAN during the study period. One patient was subsequently excluded from the study, as it represented the sole case to undergo surgery in an emergency setting. Inclusion of this patient could have introduced significant bias, given the differing perioperative circumstances and management compared with patients undergoing elective procedures.

Patient characteristics are reported in Table 1. Briefly, 32 patients were women (71%) with a median age of 55 years. All patients were high-risk and complex, exhibiting the following characteristics:

- All of them were overweight or obese, with a median maximum BMI of 40.26 kg/m^2^ and 31.79 kg/m^2^ after pre-optimisation (23% of them received bariatric surgery).
- The majority of the patients had several comorbidities, including hypertension (HTA), diabetes mellitus, and chronic obstructive pulmonary disease (COPD).
- Regarding surgical history, 47% had undergone previous VHR.
- The median length and width of the incisional hernias were 12.0 cm and 8.4 cm, respectively.
- Despite the presence of relatively small defects in some patients, the complexity of the cohort was primarily related to patient-related and anatomical factors—including obesity, loss of domain, prior repairs, and VHWG grade—rather than defect width alone.
- LOD was identified in 42% of patients and was measured according to the Tanaka volumetric method; these patients were prepared with BTA and PPP. A parastomal hernia was present in 4% of the patients.
- Regarding the risk of complications according to the modified VHWG classification, more than 70% of patients were grade 2 or 3.

**TABLE 1:** Patient demographics and characteristics.

N = 45	N (%)	Median (IQR 25–75)
Sex (women)	32 (71)	
Age (years)		55 (45–67)
Maximum BMI (kg/m^2^)		40.26 (32.6–55.63)
BMI at J0 (kg/m^2^)		31.79 (27.31–37.81)
Comorbidities HTA Diabetes COPD Active smoker Renal insufficiency	18 (40) 12 (27) 11 (24) 8 (17) 2 (4)	
Hernia history Previous hernia repair Existing mesh	21 (47) 13 (29)	
Hernia characteristics Incisional hernia Length (cm) Width (cm) LOD Parastomal	41 (91) 19 (42) 2 (4)	12 (7.8–14.7) 8.4 (5.4–12.3)
Modified VHWG 1 2 3	12 (27) 16 (36) 17 (38)	

Surgical data are reported in Table 2. All patients had total fascial closure of the abdominal wall with mesh reinforcement in 44 out of 45 cases. Long-term absorbable synthetic mesh was the most commonly used material, reflecting standard practice at our unit, and it was placed in the sublay plane. CST was performed in 19 patients, predominantly with TAR. The median operative time was 180 min and the median weight of the panniculus was 821 g. Three types of PAN incision were performed: vertical (18, 40%), horizontal (16, 36%) and *Fleur-de-Lis* (11, 24%). No prophylactic negative-pressure wound therapy was used. In three cases, the repair involved a unilateral TAR on one side, along with a contralateral Ramirez, to achieve adequate closure.

**TABLE 2:** Surgical data.

N = 45	N (%)	Median (IQR 25–75)
Fascial closure	45 (100)	
Mesh type and plane Biosynthetic Permanent Sublay	44 (98) 36 (80) 8 (18) 44 (98)	
CST TAR Ramirez TAR + Ramirez	19 (42) 13 (29) 7 (16) 3 (7)	
Type of PAN Vertical Horizontal Fleur de Lys	18 (40) 16 (36) 11 (24)	
Weight of the panniculus (g)		821 (650–1200)
Duration of surgery (min)		180 (145–211.5)
Surgical drains Subcutaneous In mesh space	42 (93) 38 (84) 6 (13)	

Concerning postoperative outcomes (Table 3), a total of 26 patients (58%) developed SSOs, predominantly scar dehiscence, primarily due to fat necrosis, and seromas, with a median healing time of 8 weeks. Deep SSIs were observed in 3 (7%) patients. Seven patients (15%) required reoperation, for wound healing purposes; they all received negative pressure wound therapy (NPWT). The median hospital length of stay was 7 days.

**TABLE 3:** Postoperative data.

N = 45	N (%)	Median (IQR 25–75)
SSO Scar dehiscence Seroma Skin necrosis Haematoma	26 (58) 17 (38) 12 (27) 5 (11) 3 (7)	
SSI	3 (7)	
Reoperation	7 (15)	
Clavien-Dindo 0 1 2 3a 3b 4 5	22 (47) 7 (16) 4 (9) 2 (4) 7 (16) 3 (7) 1 (2)	
Length of stay (LOS) (days)		7 (5–12)
Follow-up (weeks)		31 (13–62)
Recurrence	5 (11)	

During the available follow-up period, a clinical hernia recurrence was observed in 11% of patients, and one patient is currently awaiting a redo PAN. No patient had chronic pain. The median follow-up was 31 weeks.

One patient, with LOD and stage 3 according to the VHWG classification, died postoperatively due to a massive pulmonary embolism, despite accurate prevention measures being taken.

## Discussion

Incisional hernias remain one of the most common complications following abdominal surgery, affecting up to 30% of patients after laparotomy [3, 11]. Obesity is a major factor in the formation of incisional hernias due to increased intra-abdominal pressure and impaired connective tissue integrity [1]. This association has been consistently demonstrated in large cohorts (>12,000 patients) [31], international registries (>40,000 patients) [32, 33], systematic reviews and meta-analyses [34, 35] and healthcare databases (>500,000 patients) [7].

Given the rising prevalence of obesity [13], currently exceeding 45% of the population [12], surgeons are increasingly encountering complex incisional hernias in overweight patients, who often present with chronic comorbidities and impaired quality of life [1].

Obesity is associated not only with hernia development and complexity but is also a strong predictor of poor outcomes after VHR. Numerous studies have demonstrated the association between obesity and SSOs/SSIs [10, 36–38]. Preoperative weight loss reduces wound morbidity [10, 38] and technical difficulties [39]. Even a modest weight loss of 3% can result in a 15% reduction in SSI incidence [37]. Preoperative optimisation is therefore essential before VHR.

In obese patients undergoing VHR, excess pannus often complicates surgical exposure, particularly after weight loss. Since the first description of the technique in 1996 [40], simultaneous panniculectomy has emerged as a strategy to facilitate complex VHR [17]. By removing chronically infected or poorly perfused tissue, the combined procedure can improve exposure, decrease abdominal wall tension, and reduce wound morbidity while also enhancing aesthetic and functional outcomes [6, 17].

This retrospective study evaluated 45 high-risk, complex patients undergoing VHR-PAN. The literature offers conflicting conclusions regarding the additional morbidity associated with VHR-PAN *versus* VHR alone, including wound complications, operative time, and recurrence [6].

For hernia surgeons managing complex cases, a precise assessment of the additional morbidity of performing a panniculectomy concurrently with a VHR would be very helpful to improve pre- and perioperative decision-making and inform patients to facilitate voluntary consent during the pre-operative planning [13].

### Wound Morbidity

In the present article, we reported an acceptable SSO rate of 58% after VHR associated with PAN, primarily including minor events such as seromas and wound dehiscence in 27% and 38% of patients, respectively. We also reported a 7% SSI rate and a 15% reoperation rate. This mirrors findings from other studies [18, 41] that reported an overall SSO rate ranging from 34.2% to 57% in patients undergoing combined VHR and PAN, including 15% of wound dehiscence, 12% seromas, and 7% SSIs. However, the literature provides conflicting evidence concerning the increased risk of wound complications after concurrent panniculectomy with VHR. Indeed, the combination of two major operations, implying longer operative times and greater demands on wound healing compared to VHR alone, may increase the risk of complications [13]. However, this risk has not been clearly defined in the literature. Some studies have reported similar complication rates, from 17% to 30%, after VHR-PAN compared to VHR alone [6, 19, 20] and similar LOS, approximately 5.5 days. Other studies have reported higher SSO rates after the combined procedure [13, 17, 18, 42]; these included research that analysed 55,537 patients from the American Hernia Database, reporting a doubled risk of wound complications (OR = 1.69) and unplanned reoperations (OR = 2.08) [13]. Another study reported results very close to those obtained in the present study: a 57% SSO rate in VHR-PAN patients *versus* 40% in VHR alone, although the rates of severe complications requiring intervention were similar [18]. Finally, the potential disadvantages of the simultaneous panniculectomy and VHR have been a matter of debate, with conflicting evidence. A 2020 systematic review and meta-analysis showed reduced wound morbidity [21], but a more recent review, 5 years later, reported increased risks of wound morbidity and reoperation with a prolonged hospital stay [17].

The present analysis includes only complex patients and reflects the surgical activity of a dedicated and specialised hernia centre. Although the median BMI of the included patients of 32 kg/m^2^ is similar to that reported by other studies [17, 21], our work included 42% LOD, 4% parastomal hernias, and 84% of patients were grade 2 or 3 on the modified VHWG classification [30]. Both LOD and contaminated cases are independent predictors of morbidity [43] and the expected SSO rate was thus more than 50%, which is consistent with the result of 58%. None of the available studies, including the latest meta-analysis, reported such a high rate of complexity [17, 21]. A work with a very close methodology [1] to ours was recently published, including patients operated on at a high complexity centre in Bogotá, reporting surgical results after VHR-PAN. Only half of the patients were obese, only 2% of the cases were contaminated, and less than 4% were LOD. Similarly, the team at a well-known tertiary referral hernia centre in Charlotte, USA, reported their results in 2021 and 2024 [14, 24]. They included fewer than 30% contaminated cases and no LOD cases. To the best of our knowledge, we would argue that the present article probably includes the most complex patients of all the studies available reported to date. This series supports the safety, efficacy, and feasibility of this combined approach for high-risk and complex patients with acceptable complication rates. We believe that the wound morbidity resulting from adding PAN to VHR has only an anecdotal impact. Indeed, previous work has demonstrated that 71% of complex patients developed cellulitis under their pannus after open VHR without panniculectomy, with 14% requiring salvage panniculectomy secondary to wound complications [14]. As such, removing excess pannus in high-risk, complex obese patients may not only be required for technical or cosmetic reasons but may also be appropriate for achieving a durable repair.

### Recurrence

Many comparative studies have reported similar recurrence rates after VHR-PAN *versus* VHR alone after 2 years of follow-up [6, 13, 14, 19, 21, 22, 24]. In the VHR-PAN groups of these studies, recurrence rates varied from 8% to 17%. We reported an 11% recurrence rate after 31 weeks. These results raise questions:

Given that the recurrence rates can exceed 45% for complex patients undergoing VHR in certain studies [1], the good results reported in the available literature on VHR-PAN confirm that these complex patients should be managed by expert surgeons in dedicated centres [1, 14, 16].

Since the same studies reported higher rates of SSOs in the VHR-PAN groups *versus* the VHR-alone groups, the similar or lower [17, 43] recurrence rates in the VHR-PAN groups are surprising. Indeed, SSOs are known to be an important and independent risk factor of recurrence. These results may be explained by at least two factors. First, SSOs after VHR-PAN are more likely to be superficial, with no long-term negative consequences on recurrence rates. Indeed, in the present article, we reported a 58% SSO rate, including only 7% deep SSIs. Other studies have reported similar outcomes [13, 18, 24]. Second, obesity can be considered to be excess subcutaneous adipose tissue (SAT) or visceral adipose tissue (VAT). The comorbidities associated with obesity are most likely associated with excess VAT [44], and excess SAT is therefore considered a problem for plastic surgery with a lesser health impact. Indeed, patients with higher VAT volumes experience significantly more hernia recurrences and deep SSIs compared to patients with excess SAT [45]. Obese patients eligible for PAN often have excess SAT, which explains the relatively low postoperative deep SSI/SSO rates with only superficial wound complications requiring prolonged superficial dressings.

Overall, we argue that the discrepancy in outcomes may reflect differences in patient selection (primarily SAT excess and patient-specific factors, particularly wound class), surgical technique and expertise, all of which critically influence outcomes.

### QOL

Quality of life (QoL) was not evaluated in the present study; however, this aspect remains of particular interest, as the ultimate goal of this surgical strategy is to achieve both functionality and aesthetics [19]. The majority of the available literature does not adequately evaluate this issue [6]. Some studies have reported similar long-term QoL outcomes for VHR-PAN compared to VHR alone [6, 17], but others have demonstrated the benefits of this combined, single-stage procedure in restoring dynamic abdominal wall functionality and improving patients’ aesthetic appearance [1, 18]. Hutchison et al. [6] reported a 61% net improvement in QoL with VHR-PAN *versus* 36% with VHR alone.

This study has several limitations. First, its retrospective, single-centre design inherently limits the level of evidence and may introduce selection bias. Second, the relatively small sample size and the absence of a control group restrict the statistical power of the analysis. Third, the follow-up period was limited, which may result in an underestimation of the true recurrence rate. Finally, the lack of a standardised quality-of-life assessment prevents the evaluation of the functional and aesthetic impact of the combined procedure.

Despite these limitations, our study has several notable strengths. It reports the real-life experience of a high-volume tertiary referral centre specialising in complex abdominal wall reconstruction, providing valuable insight into daily surgical practice. The inclusion of a homogeneous cohort operated on by an experienced multidisciplinary team ensures consistency in surgical technique and perioperative management. Furthermore, the detailed collection of data and the systematic reporting of postoperative outcomes contribute to the robustness and transparency of the results.

### Conclusions

The safety of concurrent panniculectomy during ventral hernia repair remains a widely debated topic [18]. Patients eligible for PAN associated with VHR may present greater challenges, since obesity has been shown to increase wound complications and complexity [24]. In the present study, all patients were not only all obese but also highly complex, with more than 42% having LOD and more than 70% being stage 2 or 3 according to the modified VHWG classification. We demonstrated, in line with published literature, the feasibility, safety and efficacy of the combined procedure, which resulted in a few deep SSI complications and acceptable long-term recurrence rates. When applied to appropriately selected patients, we believe that the complication rates for this high-risk population are so high that the added wound morbidity from the PAN does not affect the surgical results and may restore the dynamic functionality of the abdominal wall with aesthetic and psychosocial benefits.

The management of these cases requires dedicated referral centres, implying at least expert surgical teams, plastic surgeons, physiotherapists, and dieticians in a multidisciplinary setting. It also requires the efforts of the patients themselves to align the interests of all parties involved. Therefore, additional research is needed to implement standardised management protocols for these complex patients, and to move towards comprehensive assessments of clinical practice and outcomes, costs, and long-term QoL after this combined reconstructive procedure. Prospective comparative studies are required to better define the impact of panniculectomy.

## Data Availability

The raw data supporting the conclusions of this article will be made available by the authors, without undue reservation.
